# Task-specific ionic liquid and CO_2_-cocatalysed efficient hydration of propargylic alcohols to α-hydroxy ketones†
†Electronic supplementary information (ESI) available. See DOI: 10.1039/c5sc00040h



**DOI:** 10.1039/c5sc00040h

**Published:** 2015-01-15

**Authors:** Yanfei Zhao, Zhenzhen Yang, Bo Yu, Hongye Zhang, Huanjun Xu, Leiduan Hao, Buxing Han, Zhimin Liu

**Affiliations:** a Beijing National Laboratory for Molecular Sciences , Key Laboratory of Colloid, Interface and Thermodynamics , Institute of Chemistry , Chinese Academy of Sciences , Beijing 100190 , China . Email: liuzm@iccas.ac.cn

## Abstract

The hydration of propargylic alcohols is a green route to synthesize α-hydroxy ketones. Herein a CO_2_-reactive ionic liquid (IL), [Bu_4_P][Im], was found to display high performance for catalyzing the hydration of propargylic alcohols in the presence of atmospheric CO_2_, and a series of propargylic alcohols could be converted into the corresponding α-hydroxy ketones in good to excellent yields. In the IL/CO_2_ reaction system, CO_2_ served as a cocatalyst by forming α-alkylidene cyclic carbonates with propargylic alcohols, and was released *via* the rapid hydrolysis of the carbonates catalysed by the IL. This is the first example of the efficient hydration of propargylic alcohols under metal-free conditions.

## Abstract 2

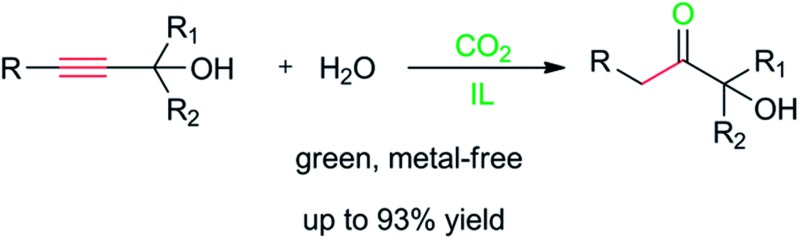
Task-specific ionic liquid and CO_2_-cocatalysed efficient hydration of propargylic alcohols to α-hydroxy ketones.

## Introduction

The selective addition of water to unsaturated bonds is of paramount importance in the production of building blocks for the synthesis of specialty chemicals.^1^ Especially the hydration of acetylenic compounds, involving simple addition of a water molecule with 100% atom efficiency for generating carbonyl compounds, has received much attention in the past decades,^2^ notably with the requirements of green chemistry and sustainable development. The key issue of the hydration of alkynes relies on the effective activation of the carbon–carbon triple bond, followed by the rapid addition of a water molecule. So far, a variety of metal (*e.g.*, Fe, Au, Ag, Ru)-based catalysts have been developed to replace traditional toxic Hg(ii) catalysts for the hydration of alkynes, where cocatalysts such as strong acids and organic ligands are generally required and/or side reactions including the Meyer–Schuster and Rupe rearrangements usually occur in these catalytic systems.^3^ The hydration of propargylic alcohols is an efficient and green route to produce α-hydroxy ketones, which are important building blocks for more elaborate molecules;^3a–c,4^ however, it is very difficult to do this under mild and metal-free conditions. Recently, Qi *et al.* reported a CO_2_-promoted route for the hydration of propargylic alcohols to α-hydroxy ketones using silver acetate (Ag_2_COO) and 1,8-diazabicyclo[5.4.0]undec-7-ene (DBU) as the catalysts at 120 °C and under high CO_2_ pressure.^5^ Our literature survey indicates that there is no report on the hydration of propargylic alcohols to α-hydroxy ketones under metal-free conditions.

Ionic liquids (ILs), possessing unique features such as high thermal and chemical stability, negligible vapor pressure, and tunable properties, have been applied in many areas.^6^ Particularly, task-specific ILs have displayed superior performances in catalysis (*e.g.*, hydrolysis reactions, CO_2_ conversion) and gas capture *via* the careful design and selection of novel component ions to endow unique properties upon them.^6f,g,7^ For example, CO_2_-reactive ILs have shown excellent performances for the capture and conversion of CO_2_ under mild conditions.^6b,7a^ The supported basic IL [Emim][HCO_3_^–^] exhibits a high activity for catalyzing the hydrolysis of propylene carbonate to 1,2-propylene glycol.^6*f*^

**Scheme 1:**
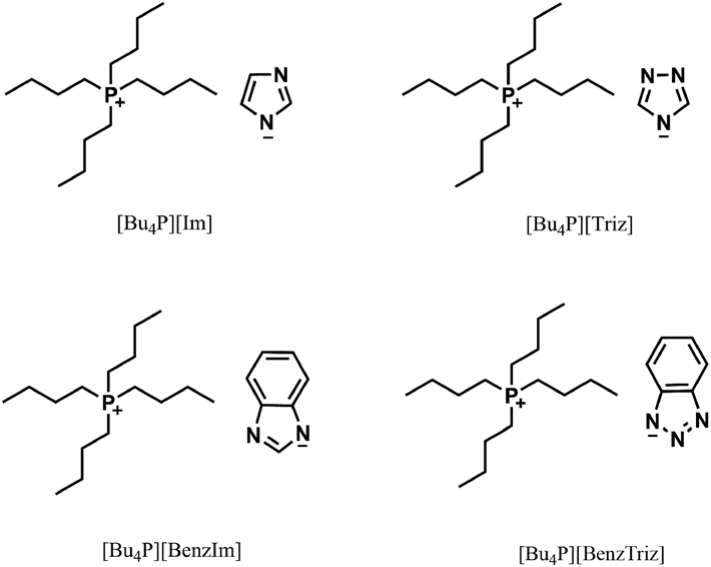
The CO_2_-reactive ILs used.

Herein, we report the hydration of propargylic alcohols to α-hydroxy ketones cocatalysed by task-specific ILs and CO_2_. The structures of all the ILs used are given in Scheme 1 and in the ESI.† It was discovered that the IL [Bu_4_P][Im] was able to catalyse the hydration of propargylic alcohols in the presence of CO_2_ at atmospheric pressure, and various propargylic alcohols could be transformed into the corresponding α-hydroxy ketones in good to excellent yields. Moreover, it was found that both CO_2_ and [Bu_4_P][Im] were indispensable for the hydration reactions, and they cocatalyzed these reactions efficiently. In addition, the IL catalyst could be easily recovered and reused without considerable activity loss. To the best of our knowledge, this is the first example of the efficient hydration of propargylic alcohols under mild and metal-free conditions. Meanwhile it is also the first time it has been found that CO_2_ can serve as a cocatalyst.

## Results and discussion

The hydration of 2-methylbut-3-yn-2-ol was carried out both in the absence and presence of ILs, and the results are listed in Table 1. It was demonstrated that this reaction did not occur in the absence of CO_2_ and/or [Bu_4_P][Im] (Table 1, entries 1–3). Excitingly, [Bu_4_P][Im] allowed the desired hydration reaction to proceed efficiently in the presence of CO_2_ at atmospheric pressure (Table 1, entry 4), solely producing 3-hydroxy-3-methyl-2-butanone in a yield of 92% (entry 4). For comparison, the other seven ILs, including [Bu_4_P][Triz], [Bu_4_P][BenzIm], [Bu_4_P][BenzTriz], [Bu_4_P][Br], [Bu_4_P][NO_3_], [Bu-DBU][Im], and [Bmim][Im], were examined for catalysis of this reaction. The results indicated that [Bu_4_P][Triz], [Bu_4_P][BenzIm] and [Bu_4_P][BenzTriz] were also effective (Table 1, entries 5–7), but showed lower activities compared to [Bu_4_P][Im], while the other ILs exhibited no activity. The significant differences in the chemical structures of these ILs may be responsible for their catalytic performances. [Bu_4_P][Triz], [Bu_4_P][BenzIm] and [Bu_4_P][BenzTriz], with the same cation as [Bu_4_P][Im], afforded lower product yields, following the order: [Bu_4_P][BenzTriz] < [Bu_4_P][Triz] < [Bu_4_P][BenzIm] < [Bu_4_P][Im]. This indicated that the catalytic activity of these ILs was significantly affected by their anions, and may be ascribed to the nucleophilicity of the anions of these ILs as a result of their different basicity.^7*a*^

**Table 1:** Hydration of propargylic alcohol promoted by different IL catalysts in the presence of CO_2_ ^*a*^

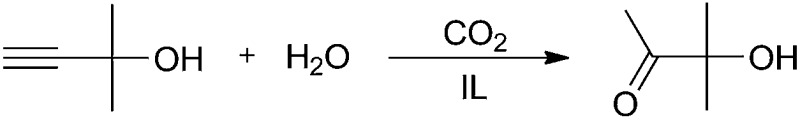				
Entry	Catalyst	CO_2_ pressure (MPa)	Anion p*K*_a_ in DMSO^7*a*^	Yield^*b*^ [%]
1	—	—	—	0
2^*c*^	[Bu_4_P][Im]	—	—	0
3	—	0.1	—	0
4	[Bu_4_P][Im]	0.1	18.6	92
5	[Bu_4_P][BenzIm]	0.1	16.4	83
6	[Bu_4_P][Triz]	0.1	13.9	82
7	[Bu_4_P][BenzTriz]	0.1	11.4	39
8	[Bu-DBU][Im]	0.1	—	0
9	[Bmim][Im]	0.1	—	0
10^*d*^	[Bu_4_P][Br]	0.1	—	0
11	[Bu_4_P][NO_3_]	0.1	—	0

^*a*^Reaction conditions: substrate (1 mmol), H_2_O (2 mmol), IL (3 mmol), 353 K, 24 h.

^*b*^Determined by ^1^H NMR ([D_6_]DMSO) using *tert*-butyl alcohol as an internal standard.

^*c*^Under N_2_ atmosphere.

^*d*^H_2_O (0.5 mL) was added to the reaction mixture.

[Bu-DBU][Im] and [Bmim][Im], with the same anion as [Bu_4_P][Im], gave no product (Table 1, entries 8 and 9), suggesting that the cations of the ILs affected the activities of the catalysts more significantly that the anion. In addition, [Bu_4_P][Br], and [Bu_4_P][NO_3_] with the same cation as [Bu_4_P][Im] also showed no activity (Table 1, entries 10 and 11), which may be ascribed to the very weak interactions between their anions and CO_2_. From the above results, it can be concluded that both CO_2_ and the task-specific ILs were indispensable, and their synergistic effects resulted in the formation of the final products. In addition, the chemical structures (both cations and anions) of the ILs played important roles in the hydration of 2-methylbut-3-yn-2-ol.

Encouraged by the above results, the generality of [Bu_4_P][Im]/CO_2_-catalyzed hydration reactions of diverse propargylic alcohols was evaluated. The results indicated that most reactions proceeded smoothly, producing corresponding α-hydroxy ketones in good to excellent yields under experimental conditions (Table 2, entries 1–15). For example, 3-methyl-1-pentyn-3-ol afforded the corresponding product in a yield of 77% within 24 h (Table 2, entry 2), comparable to that reported previously using Ag_2_COO and DBU as catalysts at 120 °C and 2 MPa of CO_2_ pressure (Table 2, entry 16).^5^ Moreover, prolonging the reaction time to 48 h, the product yield reached 93% (Table 2, entry 3). Notably, the steric effects of the substituents in the substrates had significant effects on their activities in the formation of the corresponding α-hydroxy ketones. This was confirmed by the fact that the product yields decreased when the lengths of the substituent chains in the propargylic alcohols increased (Table 2, entries 1, 2, 4, and 6). Moreover, large scale reactions of substrates including 3-methyl-1-nonyn-3-ol, 2-phenyl-3-butyn-2-ol and 2-methyl-4-phenyl-3-butyn-2-ol were carried out, and the corresponding α-hydroxy ketones were isolated in yields of 84%, 87%, and 85%, respectively (Table 2, entries 8, 12 and 15, respectively), showing that the present system has great potential for applications. In addition, 3-butyn-2-ol, 3,3-dimethyl-1-butyne and 3-chloro-3-methylbut-1-yne were also employed as substrates; however, the hydration reactions did not occur, and no products were obtained in these cases. This suggests that the presence of an –OH linked to the C next to the alkynyl C is necessary for the hydration of propargylic alcohols.

**Table 2:** Hydration of various propargylic alcohols over the [Bu_4_P][Im]/CO_2_ catalyst^*a*^

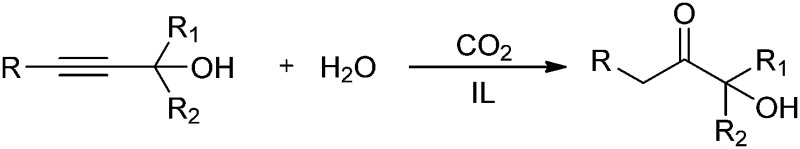				
Entry	Substrate	Time/h	Product	Yield/%
1	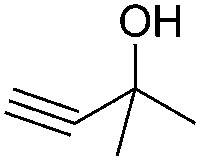	24	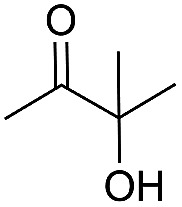	92
2	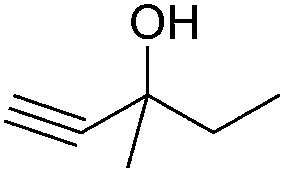	24	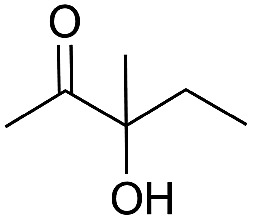	77
3		48		93
4	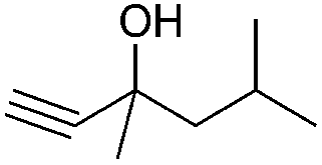	40	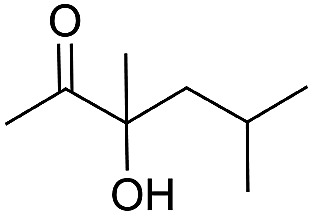	62
5		24		86^*b*^
6	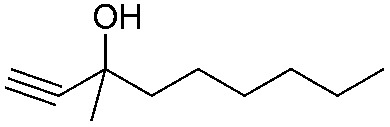	40	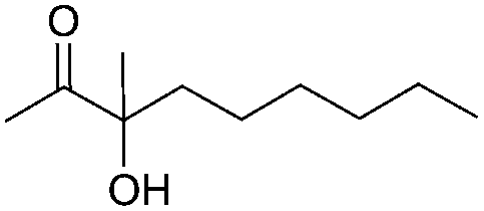	51
7		40		90^*b*^
8		40		84^*c*^
9	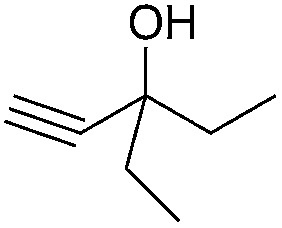	48	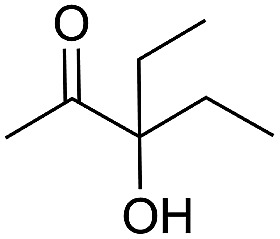	90
10	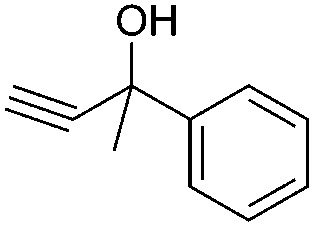	40	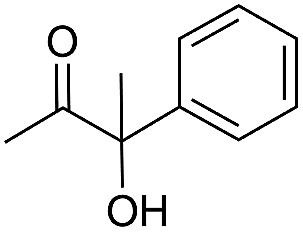	68
11		24		90^*b*^
12		24		87^*c*^
13	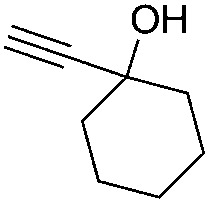	48	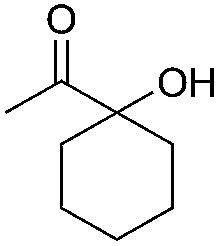	88^*b*^
14	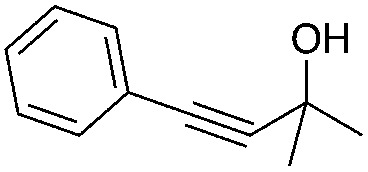	48	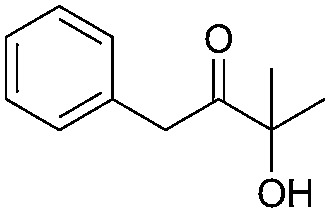	90^*b*^
15		48		85^*c*^
16	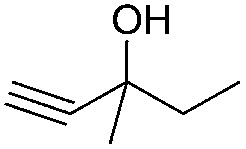	24	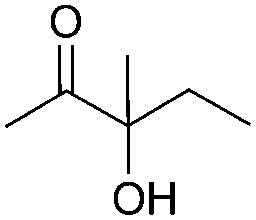	78^*d*^
17	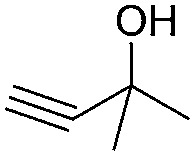	24	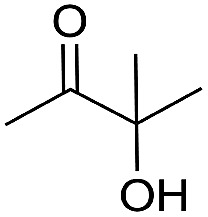	88^*e*^

^*a*^Reaction conditions: substrate (1 mmol), [Bu_4_P][Im] (3 mmol), CO_2_ (0.1 MPa), H_2_O (2 mmol), 353 K. Yield was determined by ^1^H NMR ([D_6_]DMSO) using *tert*-butyl alcohol as an internal standard.

^*b*^CO_2_ (1 MPa).

^*c*^Isolated yield, substrate (7 mmol), [Bu_4_P][Im] (21 mmol), CO_2_ (1 MPa), H_2_O (14 mmol).

^*d*^Substrate (0.5 mmol), AgOAc (10% mol), DBU (0.25 mmol), CO_2_ (2 MPa), H_2_O (0.3 mL), MeCN (1 mL), 393 K.

^*e*^[Bu_4_P][Im] was used for the fifth time.

To explore the reusability of the IL catalyst, five catalytic cycles of 2-methylbut-3-yn-2-ol hydration were performed over the [Bu_4_P][Im] catalyst in the presence of CO_2_. It was demonstrated that the product yield almost remained unchanged (Table 2, entry 17; Fig. S8†), suggesting that [Bu_4_P][Im] was stable and the designed catalytic system was recyclable.

**Fig. 1:**
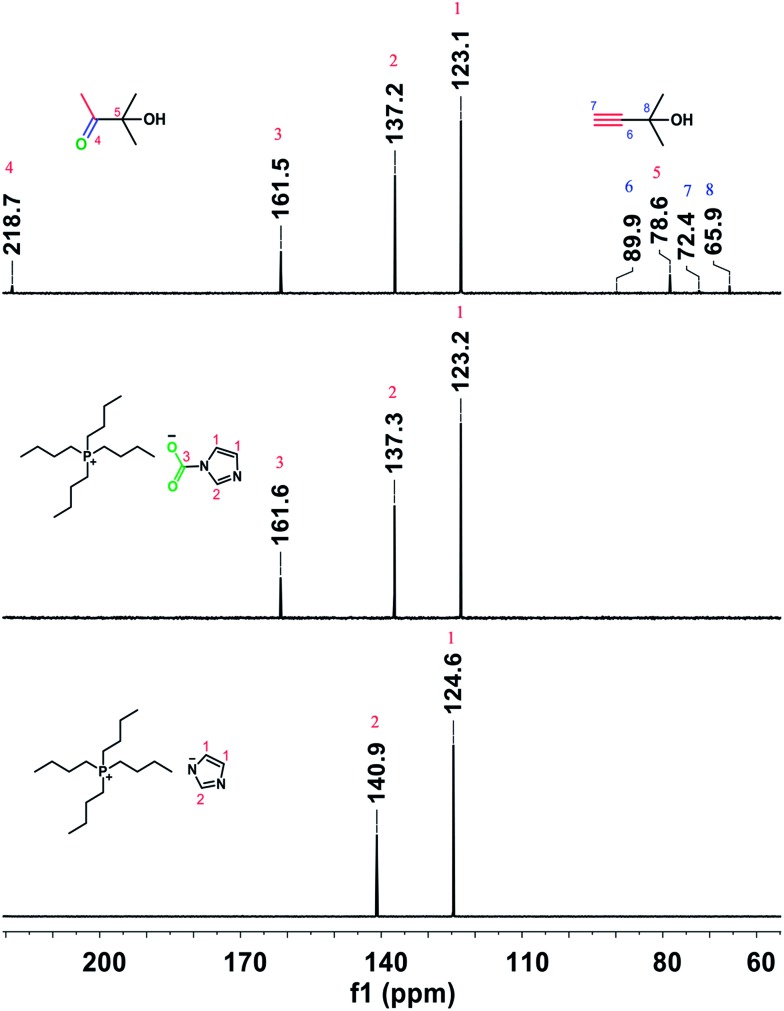
^13^C NMR spectrum of pure [Bu_4_P][Im], the intermediate of [Bu_4_P][Im] exposed to CO_2_ (0.1 MPa), and the reaction solution of 2-methylbut-3-yn-2-ol hydration in the presence of atmospheric CO_2_ at 80 °C for 7 h (D_2_O, 0.6 mL, 298 K, using DMSO as the internal standard).

The above results indicate that CO_2_ plays an important role in the hydration of various propargylic alcohols. To explore the reaction mechanism, the IL, the IL exposed to CO_2_, and the reaction solution for 2-methylbut-3-yn-2-ol hydration in the presence of atmospheric CO_2_ at 80 °C for 7 h were examined by NMR analysis. As illustrated in Fig. 1, a new signal appeared at 161.6 ppm in the ^13^C NMR spectrum of the IL exposed to atmospheric CO_2_, which was attributed to the carbonyl carbon of carbamate, indicating that CO_2_ could react with the anion [Im^–^] of the IL to form an intermediate ([Bu_4_P][ImCOO]), in agreement with previous reports.^7*a*^ This signal also appeared at 161.5 ppm in the spectrum of the reaction solution, suggesting that in the reaction process the IL could activate CO_2_ to form a [Bu_4_P][ImCOO] intermediate. The carbamate anion moiety in the [Bu_4_P][ImCOO] intermediate may attack the triple bond in 2-methylbut-3-yn-2-ol,^8*a*^ thus facilitating the conversion of this propargylic alcohol. The ^13^C NMR analysis indicated that besides 2-methylbut-3-yn-2-ol and 3-hydroxy-3-methyl-2-butanone, the species from CO_2_ and the anion of the IL were detectable in the reaction solution. This implies that the IL served as a catalyst for activating CO_2_ in the reaction process, and the resultant [Bu_4_P][ImCOO] intermediate played a significant role in the formation of final product. Considering that [Bu-DBU][Im] and [Bmim][Im] showed no activity for the hydration of 2-methylbut-3-yn-2-ol (Table 1, entries 8 and 9), ^13^C NMR analyses of [Bu-DBU][Im] and [Bmim][Im] exposed to atmospheric CO_2_ were performed, respectively, and no obvious peaks attributable to carbamates formed between the ILs and CO_2_ were observed in the corresponding ^13^C NMR spectra, suggesting that these two ILs could not activate CO_2_ efficiently. The steric hindrance around the P or N atoms in these ILs may be responsible for their significantly different performances for activating CO_2_. From the above findings, it can be further deduced that in the [Bu_4_P][Im]/CO_2_ reaction system the [Bu_4_P][ImCOO] intermediate was crucial for the synthesis of the final product.

It was reported that CO_2_ could react with propargylic alcohols to produce α-alkylidene cyclic carbonates catalyzed by metal and base catalysts.^8^ The control experiment in this study demonstrated that α-alkylidene cyclic carbonate could be formed from CO_2_ and 2-methylbut-3-yn-2-ol catalyzed by [Bu_4_P][Im] with a trace amount of H_2_O at atmospheric pressure, confirmed by ^1^H NMR analysis (see Fig. S9†). However, α-alkylidene cyclic carbonates were not detectable in the reaction solutions for the hydration of various propargylic alcohols. Another control experiment, hydrolysis of α-alkylidene cyclic carbonate in [Bu_4_P][Im], was performed. To our delight, this cyclic carbonate was rapidly hydrolysed at 80 °C within 1 h, affording 3-hydroxy-3-methyl-2-butanone in a yield approaching 100%. The above findings suggest that the α-alkylidene cyclic carbonate formed from CO_2_ reacting with 2-methylbut-3-yn-2-ol may be the key intermediate for the formation of 3-hydroxy-3-methyl-2-butanone.

**Scheme 2:**
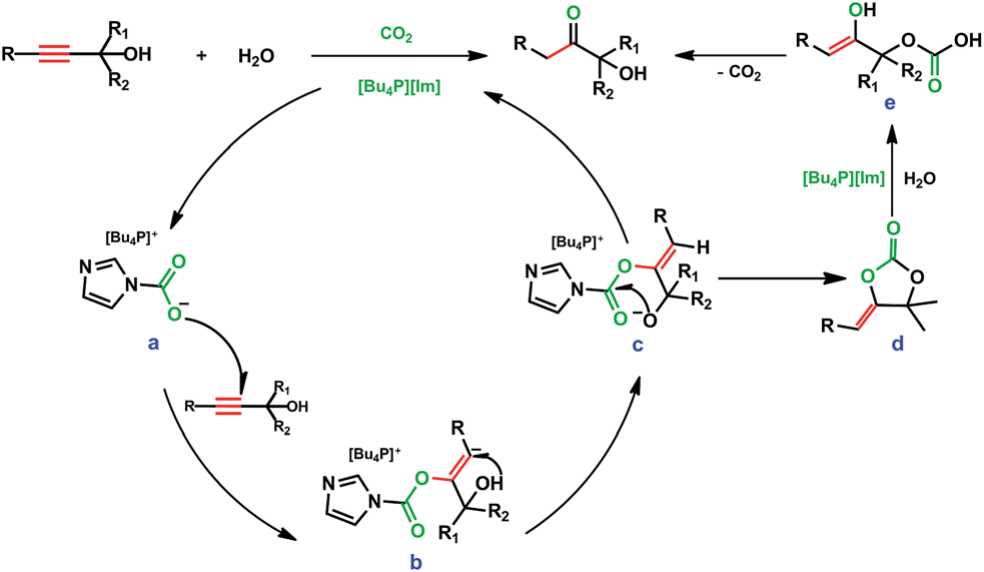
Possible reaction pathway.

On the basis of the experimental results and previous reports,^5,8,9^ a possible mechanism for IL and CO_2_-cocatalyzed hydration of propargylic alcohols was proposed, as shown in Scheme 2. CO_2_ is first activated by the anion [Im^–^] to form intermediate **a**, [Bu_4_P][ImCOO]. The nucleophilic O atom of intermediate **a** attacks the triple bond of the propargylic alcohol to form intermediate **b**, which then undergoes hydrogen migration from the hydroxyl group of the alcohol to produce intermediate **c**; the alkoxide anion of intermediate **c** attacks the carbonyl carbon to produce intermediate **d** after release of the IL; rapid hydrolysis of intermediate **d** catalysed by [Bu_4_P][Im] produces **e**, which subsequently converts to the product α-hydroxy ketone *via* keto–enol tautomerization and the release of CO_2_. In this reaction process, CO_2_ is involved in the formation of the key intermediates (*i.e.*, α-alkylidene cyclic carbonates), and is rapidly released *via* the hydrolysis of these intermediates. CO_2_ is required, but not consumed in the whole reaction process, and plays a catalyst-like role in the formation of the final product.

In addition, it should be pointed out that CO_2_ was able to react with propargylic alcohols catalyzed by [Bu_4_P][Im] in the absence of water, producing α-alkylidene cyclic carbonates at atmospheric pressure. This is a new metal-free catalytic route for the synthesis of α-alkylidene cyclic carbonates through coupling reactions of CO_2_ with propargylic alcohols under mild conditions.

## Conclusions

In summary, we have developed a green, metal-free and efficient method for the hydration of propargylic alcohols to generate α-hydroxy ketones using [Bu_4_P][Im]/CO_2_ as the catalytic system, which enables the reactions to proceed smoothly and to afford good to excellent yields of products. The IL and CO_2_ have an excellent synergistic effect on catalyzing the reactions, and CO_2_ serves as a cocatalyst. In addition, the multifunctional IL can be easily recovered and reused without obvious loss in its activity. We believe that this kind of highly-efficient and greener CO_2_–IL catalytic system has great potential for applications.
