# Supplementary material for: Task-specific ionic liquid and CO2-cocatalysed efficient hydration of propargylic alcohols to α-hydroxy ketones
Source: Chem Sci. 2015 Jan 15;6(4):2297–301. doi: 10.1039/c5sc00040h (PMC5645775; doi:10.1039/c5sc00040h)
Supplement: Supplementary file 1 [file SC-006-C5SC00040H-s001.pdf]

Electronic Supplementary Material (ESI) for Chemical Science.
This journal is © The Royal Society of Chemistry 2015

Supporting information for

Task-specific ionic liquid and CO_2 cocatalysed efficient hydration of propargylic alcohols to α-hydroxy ketones

*Yanfei Zhao, Zhenzhen Yang, Bo Yu, Hongye Zhang, Huanjun Xu, Leiduan Hao, Buxing Han and Zhimin Liu**

Beijing National Laboratory for Molecular Sciences, Key Laboratory of Colloid, Interface and Thermodynamics, Institute of Chemistry, Chinese Academy of Sciences, Beijing 100190, China.

E-mail: liuzm@iccas.ac.cn

1. Experimental Section.
2. Characterization for the as-synthesized ionic liquids, Figure S1-S7.
3. Yields of 3-hydroxy-3-methyl-2-butanone obtained using recycled $[Bu_4P][Im]$ as the catalyst, Figure S8.
4. ^{1}H NMR spectra of intermediates, Figure S9.
5. ^{1}H and ^{13}C NMR spectra of the products, Figure S10-S25.
6. References

1. Experimental Section

1.1 Materials

CO_2 was supplied by Beijing Analytical Instrument Factory with a purity of 99.99%. Various propargylic alcohol substrates were purchased from Beijing Innochem Science & Technology Co., Ltd. Tetrabutylphosphonium hydroxide ([Bu_4P][OH], 40% wt.% solution in water), tetrabutylphosphonium bromide ([Bu_4P][Br]), tetrabutylphosphonium Benzotriazolate ([Bu_4P][Bentriz]), imidazole, benzimidazole and trizole were obtained commercially from Alfa Aesar and Tokyo chemical industry Co., Ltd, respectively. Acetic acid, nitric acid and other chemicals were purchased from Beijing Chemical Company. The deuterated solvents ([D_6]DMSO, D_2O and $CDCl_3$) were provided by Cambridge Isotope Laboratories, Inc. All chemicals were of analytical grade and used as received. According to the reported procedures,[S1] [Bu_4P][Im], [Bu_4P][Triz], [Bu_4P][BenIm], [Bu_4P][Ac], [Bu_4P][NO_3] were synthesized by neutralizing corresponding base [Bu_4P][OH] and proton donors (Imidazole, trizole, benzimidazole, acetic acid and nitric acid), respectively. [Bu-DBU][Im] and [BMIM][Im] was prepared by the method similar to that reported by other authors.[S2] The designed ionic liquids were characterized by NMR techniques. ^1H and ^{13}C NMR analyses were conducted in D_2O or $CDCl_3$ on a Bruker Avance NMR (400 MHz). The characterization data of the ionic liquids were reported below (Figure S1-S7).

1.2 General procedure for hydration of propargylic alcohols

In a typical experiment, propargylic alcohol (1 mmol, 0.084 g) and [Bu_4P][Im] (3 mmol, 0.978 g) were loaded in a 10 mL flask equipped with a magnetic stirrer. The CO_2 pressure was kept at 0.1 MPa using a balloon. The reaction mixture was stirred under refluxing at 353 K for 24 h. Finally, the reaction mixture was cooled in ice water and transferred into a volumetric flask. The quantitative analysis was conducted by ^1H NMR analysis using tert-butyl alcohol as an internal standard. For large scale reactions, the corresponding products were isolated by column chromatography. The isolated yields of the products were calculated based on the masses determined by mass balance. IL was recovered by adding diethyl ether into the reaction system to extract product and reactant, and then the IL was used directly for the next run after removing solvent by rotary evaporation and drying in a vacuum oven at 343 K for 48 h to remove the residual reactants.

2. Characterization for the as-synthesized ionic liquids

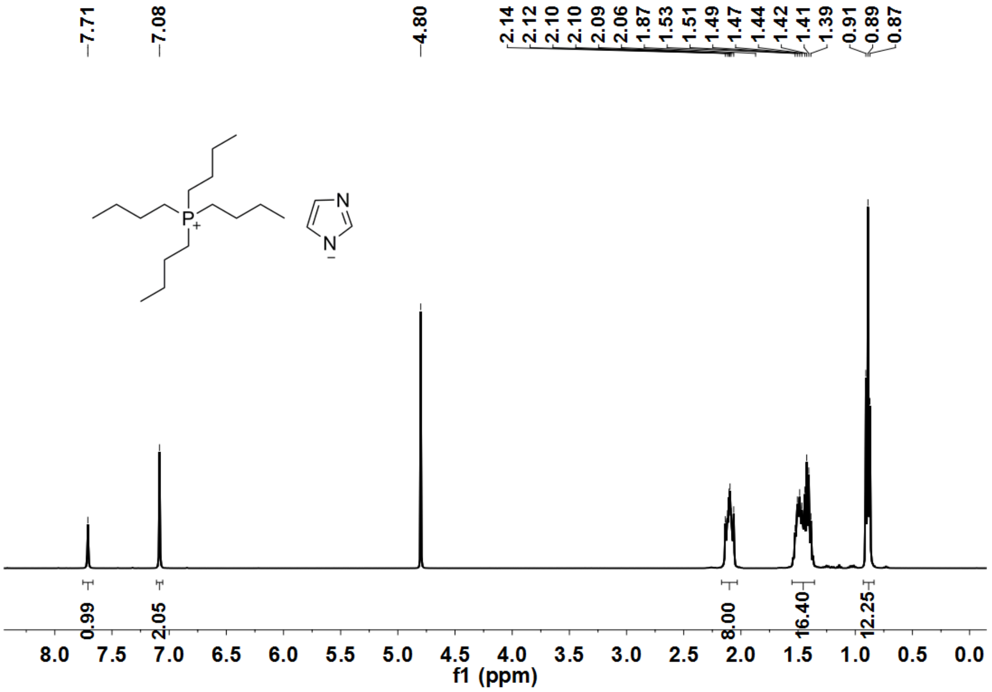

Figure S1. ^{1}H NMR spectrum of tetrabutylphosphonium imidazolide [Bu_4P][Im] (D_2O, 298 K)

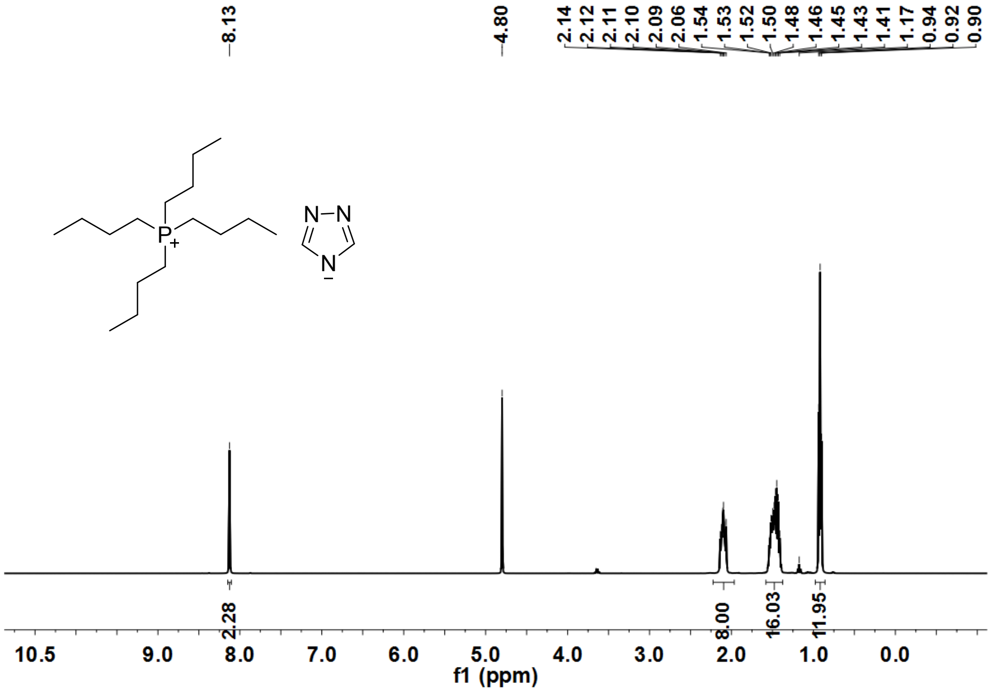

Figure S2. ^{1}H NMR spectrum of tetrabutylphosphonium 1,2,3-triazolide [Bu_4P][Triz] (D_2O, 298 K)

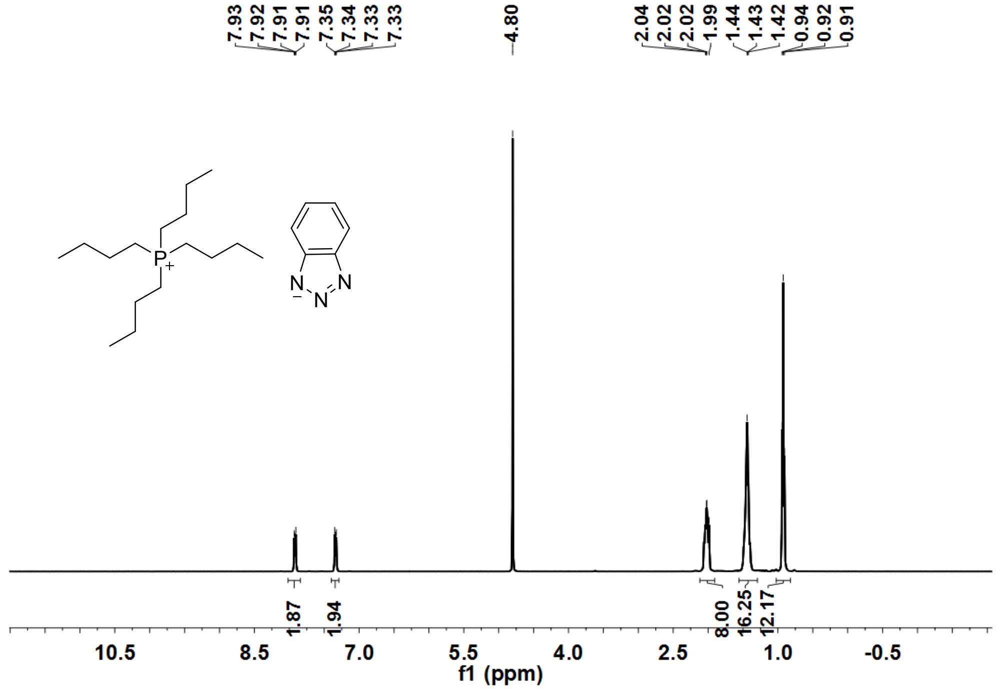

Figure S3. ^{1}H NMR spectra of tetrabutylphosphonium benzotriazolate [Bu_4P][Bentriz] (D_2O, 298 K)

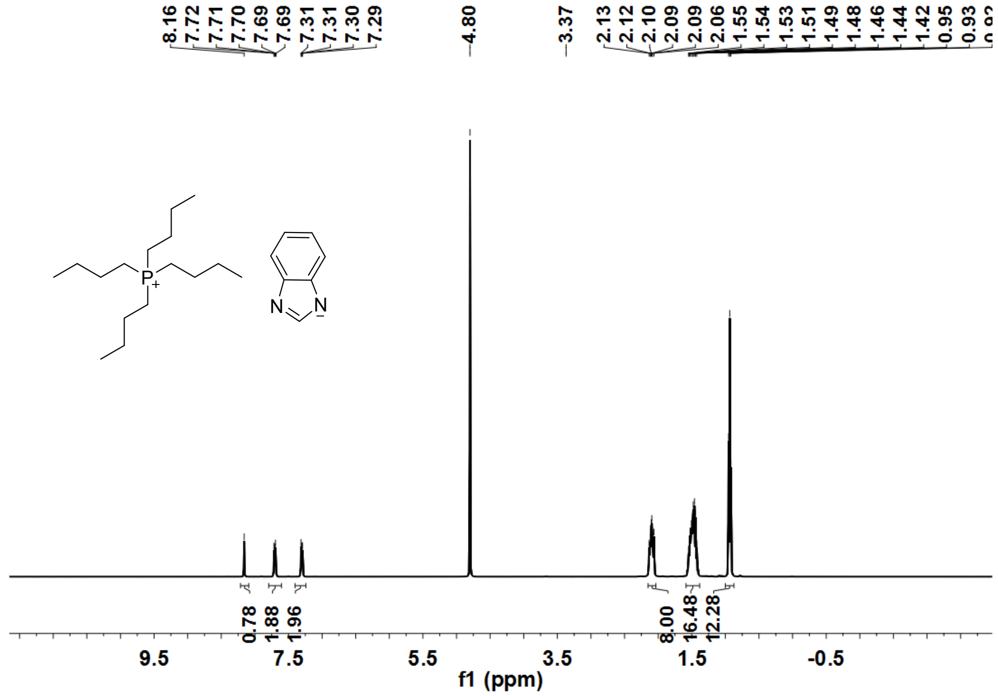

Figure S4. ^{1}H NMR spectrum of tetrabutylphosphonium benzimidazolate [Bu_4P][BenMi] (D_2O, 298 K)

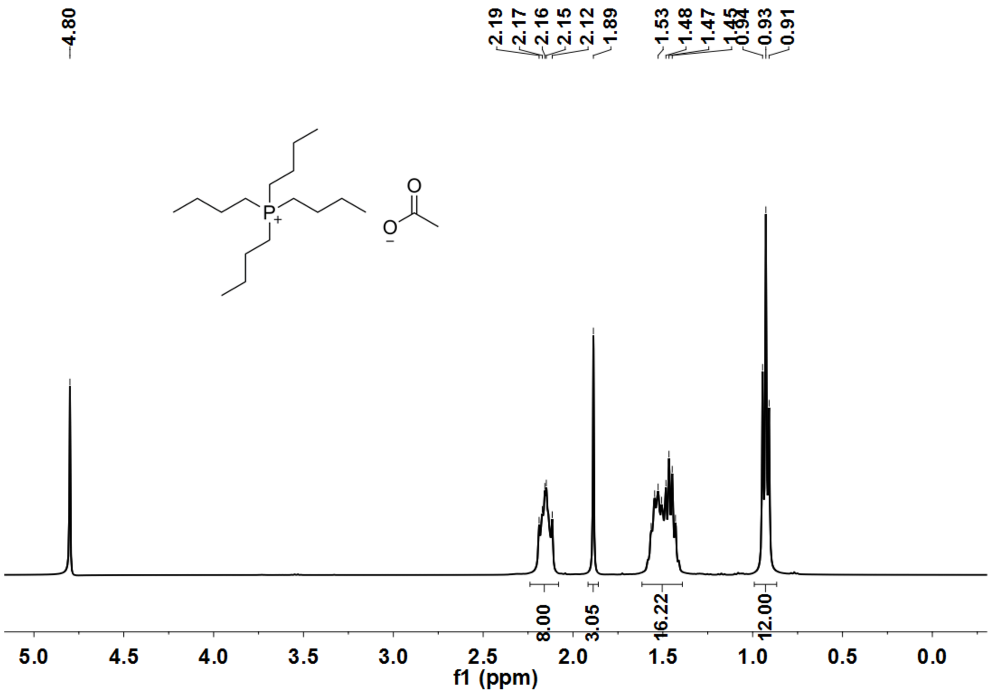

Figure S5. ^{1}H NMR spectrum of tetrabutylphosphonium acetate $[Bu_4P][Ac]$ (D_2O, 298 K)

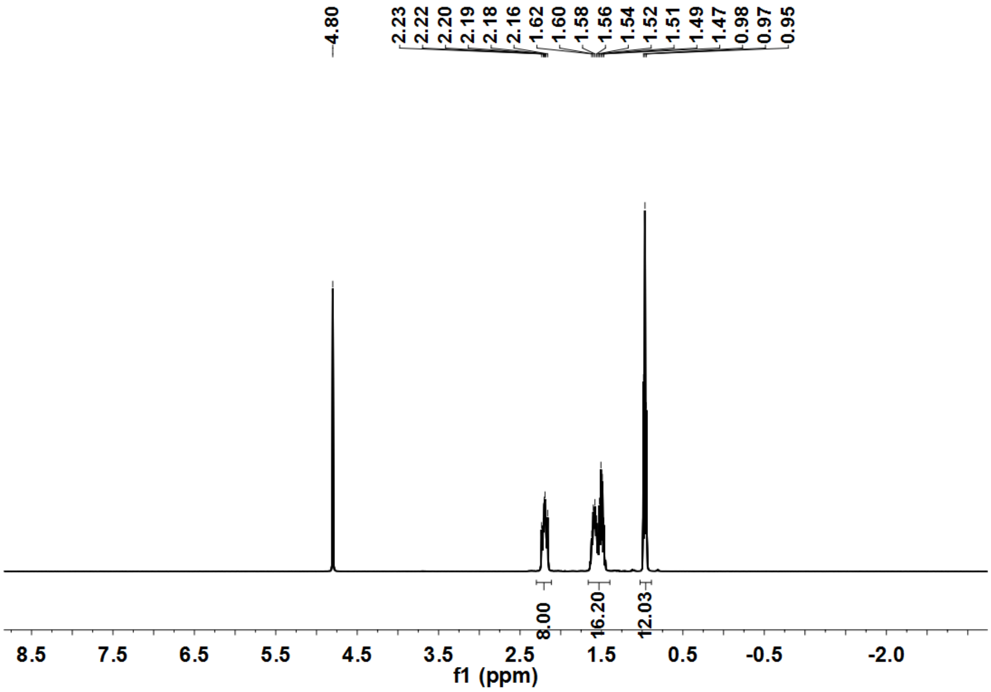

Figure S6. ^{1}H NMR spectrum of tetrabutylphosphonium nitrate $[Bu_4P][NO_3]$ (D_2O, 298 K)

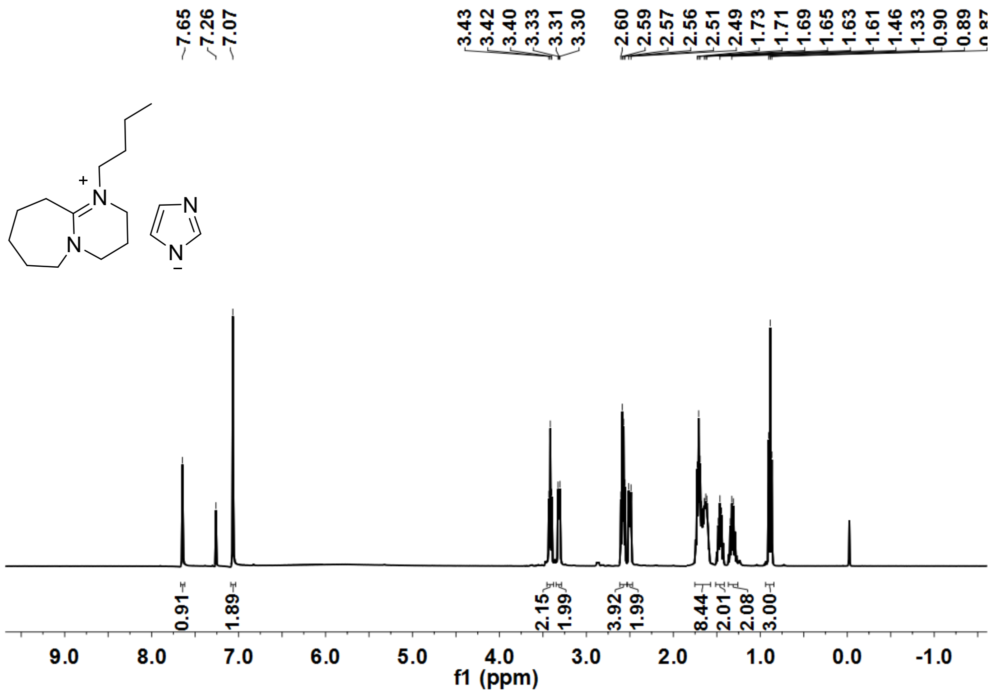

Figure S7. ^{1}H NMR spectrum of 1-butyl-2,3,4,6,7,8,9,10-octahydropyrimido[1,2-a] azepin-1-ium imidazolide [Bu-DBU][Im] ($CDCl_3$, 298 K)

3. Yields of 3-hydroxy-3-methyl-2-butanone obtained using recycled [Bu_4P][Im] as the catalyst

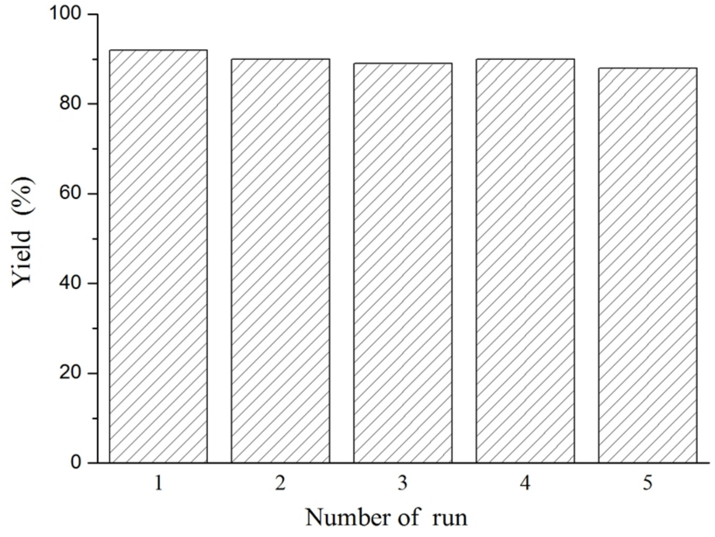

Figure S8. Yields of 3-hydroxy-3-methyl-2-butanone obtained using recycled [Bu_4P][Im] as the catalyst

4. ^{1}H NMR spectra of intermediates

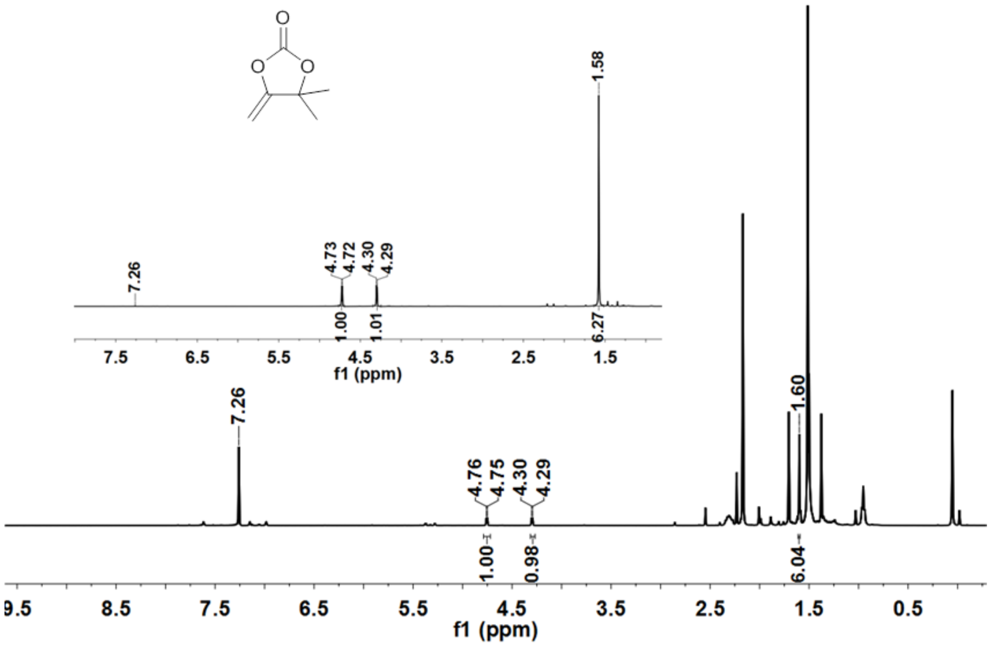

Figure S9. ^{1}H NMR spectra of control experiment ($CDCl_3$, 298K)

5. ^{1}H and ^{13}C NMR spectra of the products ([D_6]DMSO, 298K)

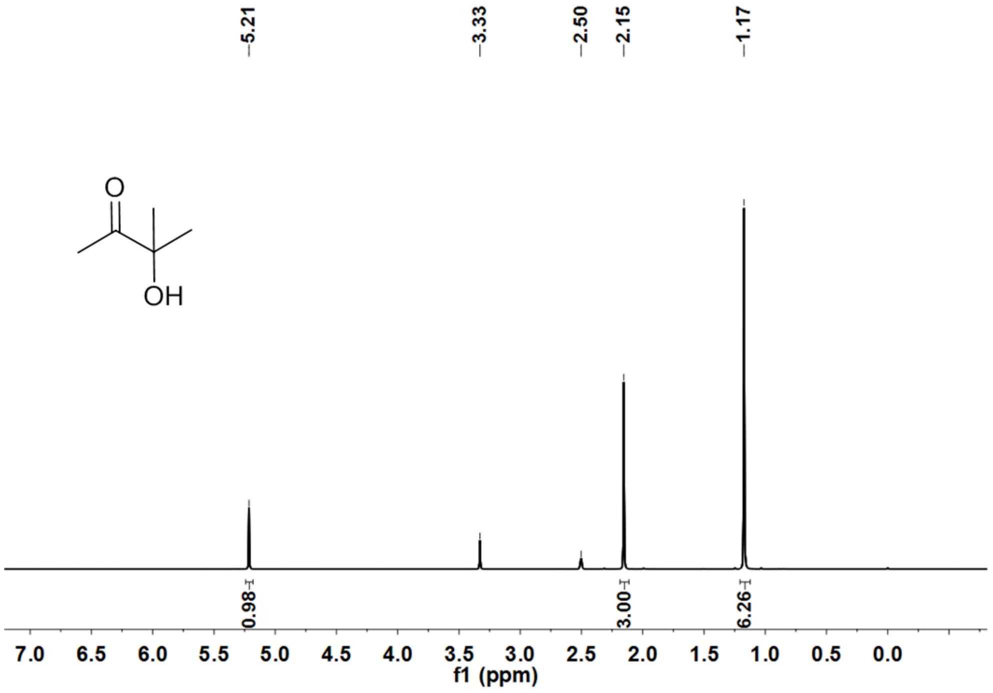

Figure S10. ^{1}H NMR spectrum of 3-hydroxy-3-methyl-2-butanone

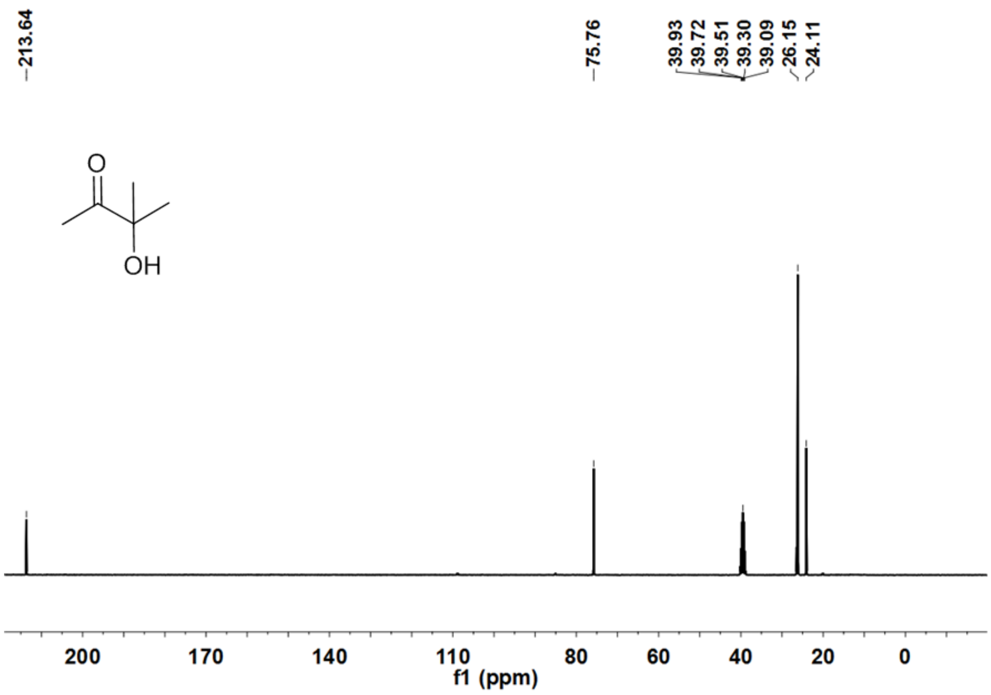

Figure S11. ^{13}C NMR spectrum of 3-hydroxy-3-methyl-2-butanone

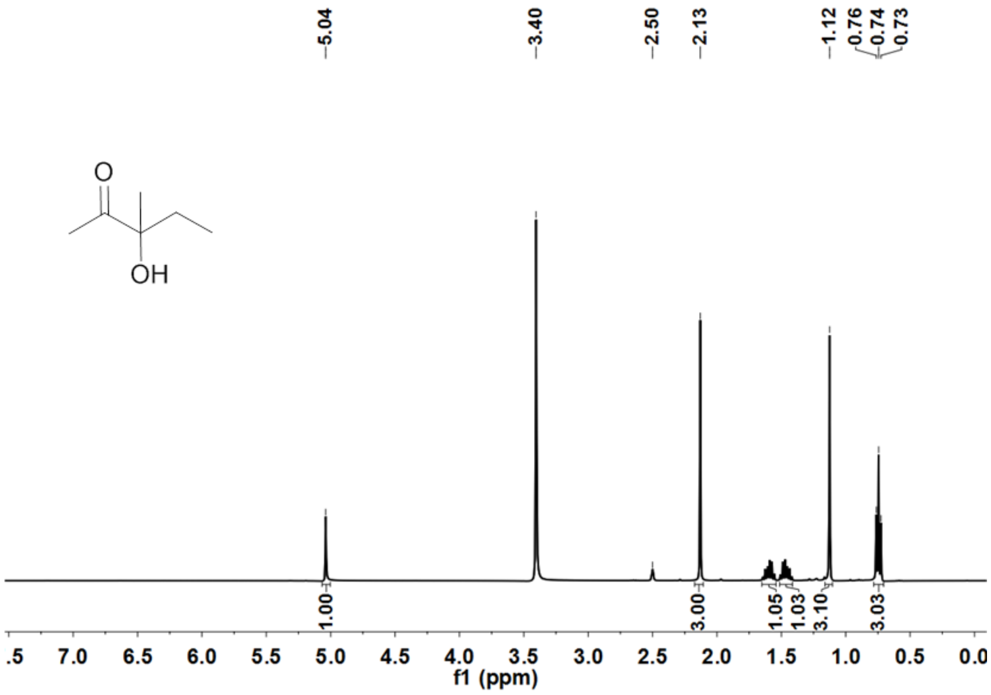

Figure S12. ^{1}H NMR spectrum of 3-hydroxy-3-methyl-2-pentanone

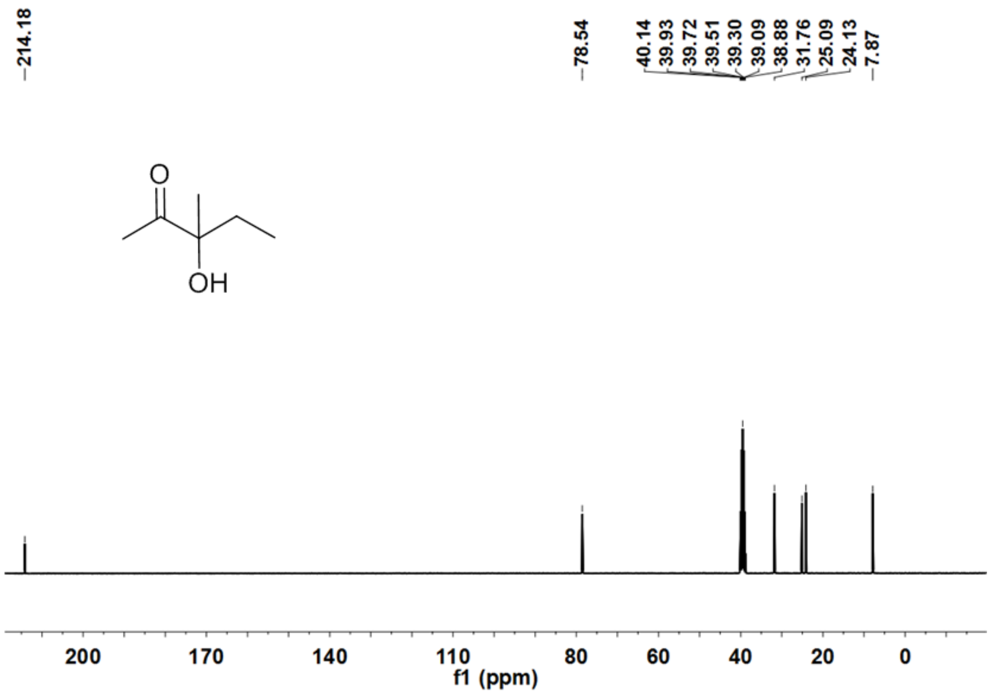

Figure S13. ^{13}C NMR spectrum of 3-hydroxy-3-methyl-2-pentanone

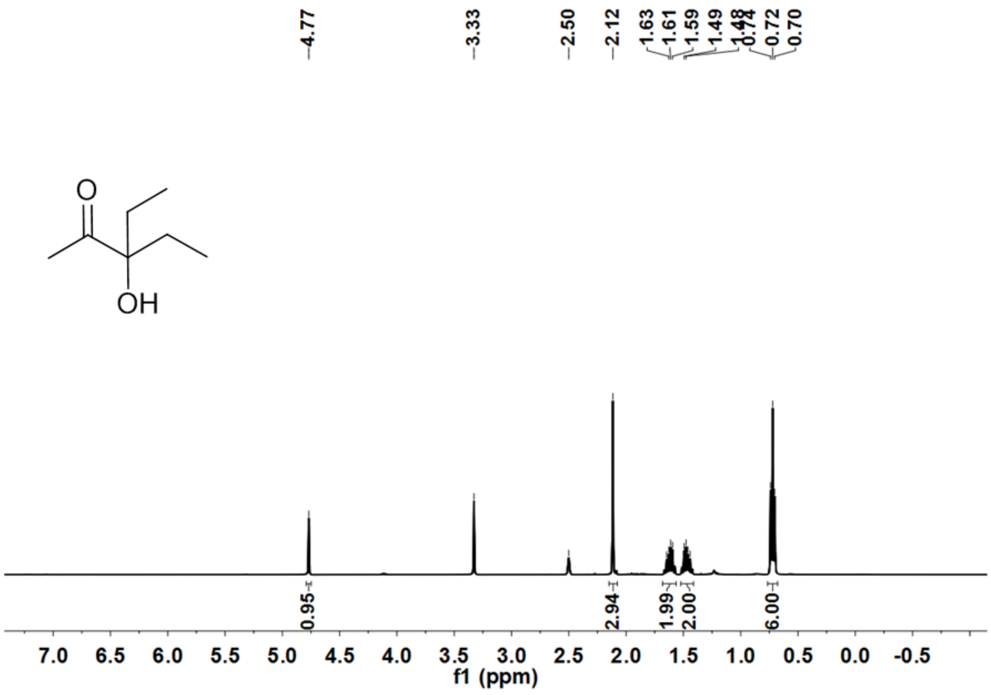

Figure S14. ^{1}H NMR spectrum of 3-ethyl-3-hydroxy-pentan-2-one

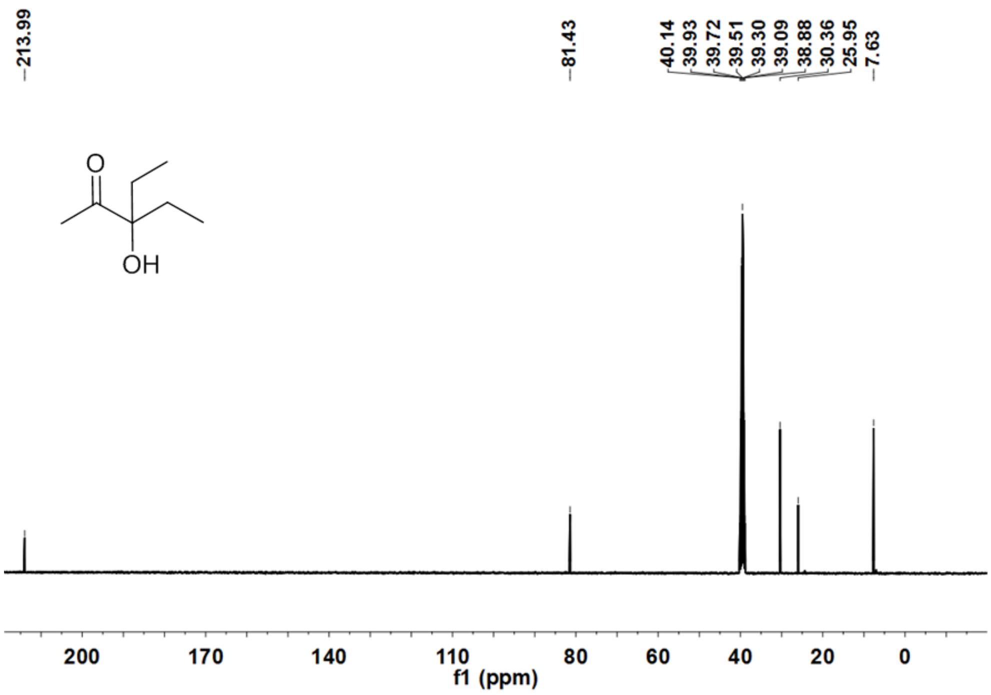

Figure S15. ^{13}C NMR spectrum of 3-ethyl-3-hydroxy-pentan-2-one

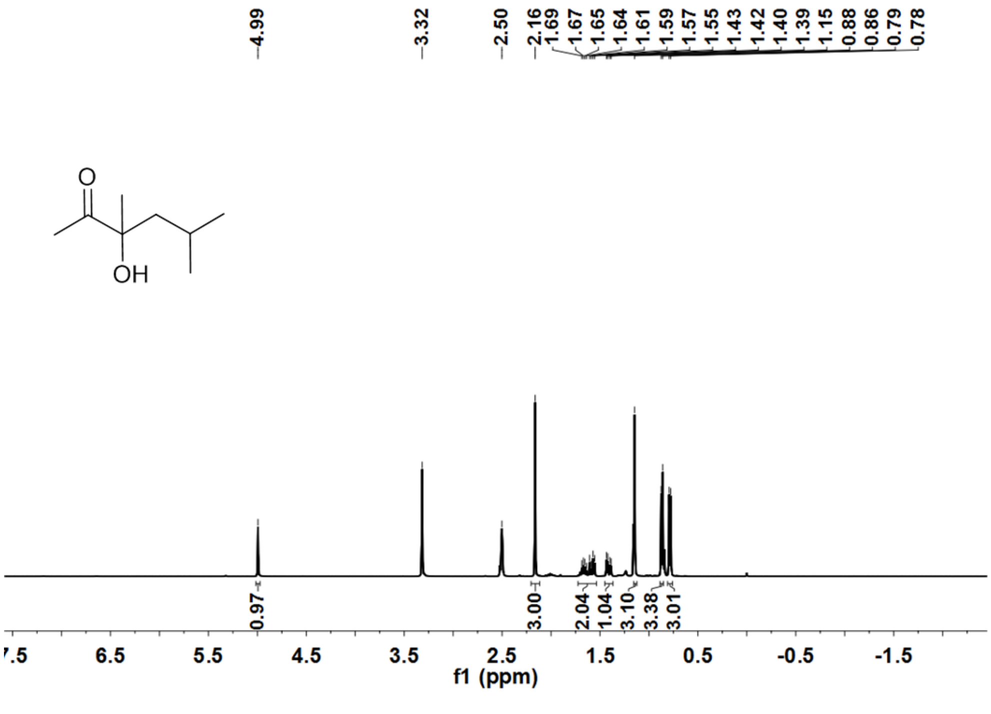

Figure S16. ^{1}H NMR spectrum of 3-hydroxy-3,5-dimethyl-2-hexanone

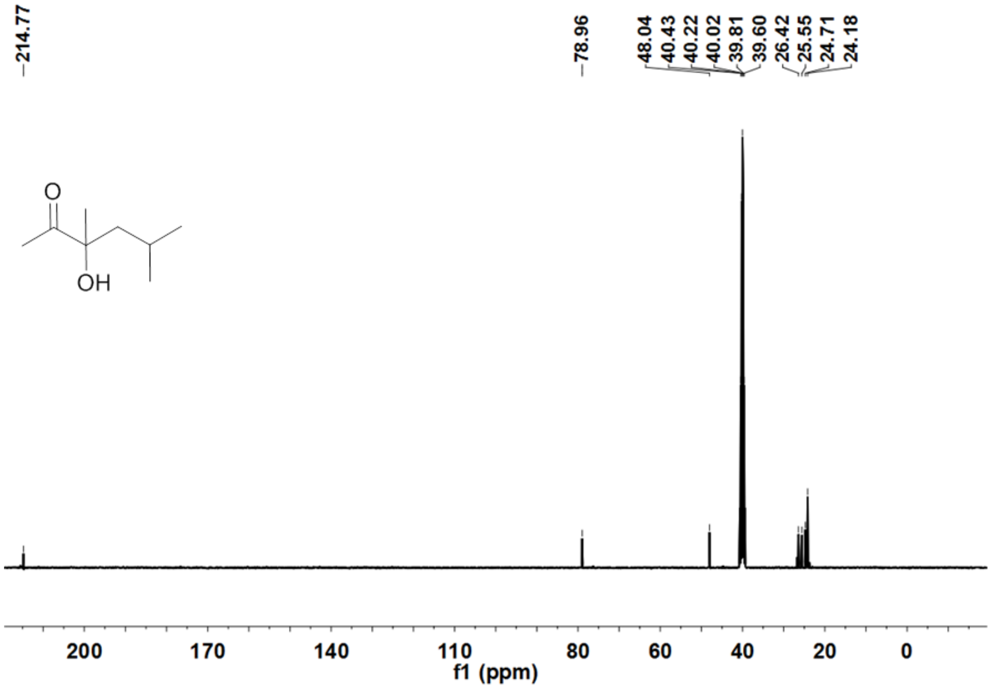

Figure S17. ^{13}C NMR spectrum of 3-hydroxy-3,5-dimethyl-2-hexanone

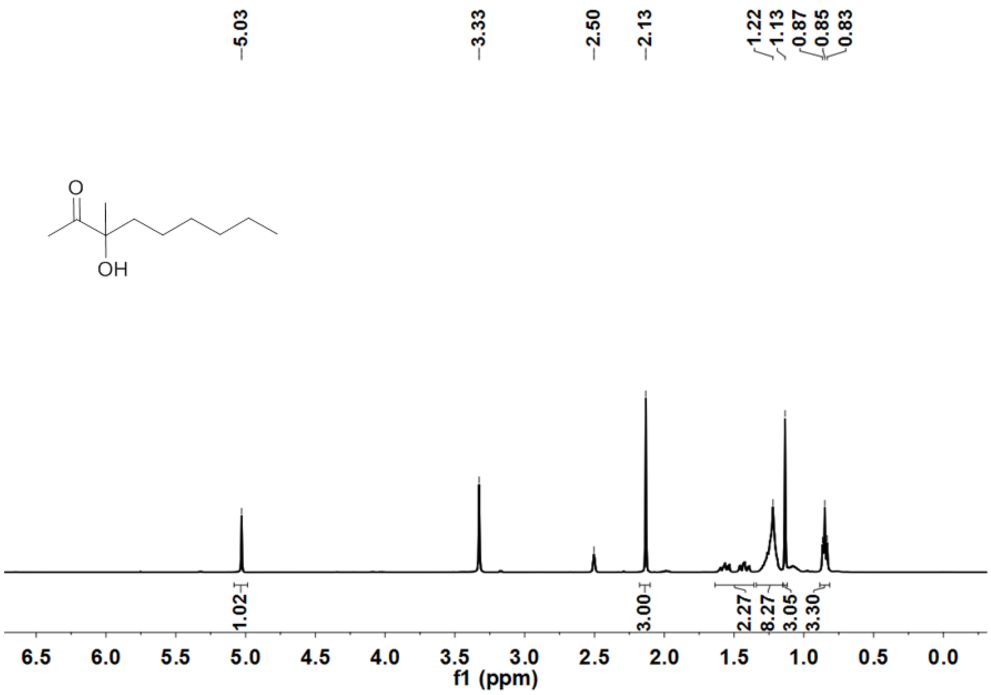

Figure S18. ^{1}H NMR spectrum of 3-hydroxy-3-methyl-2-nonanone

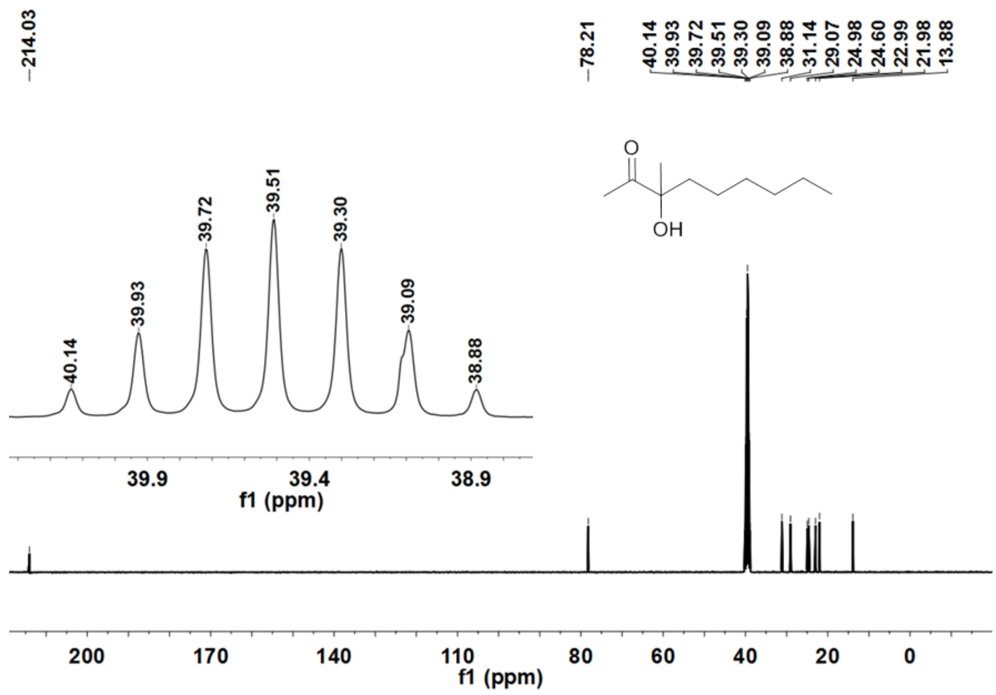

Figure S19. ^{13}C NMR spectrum of 3-hydroxy-3-methyl-2-nonanone

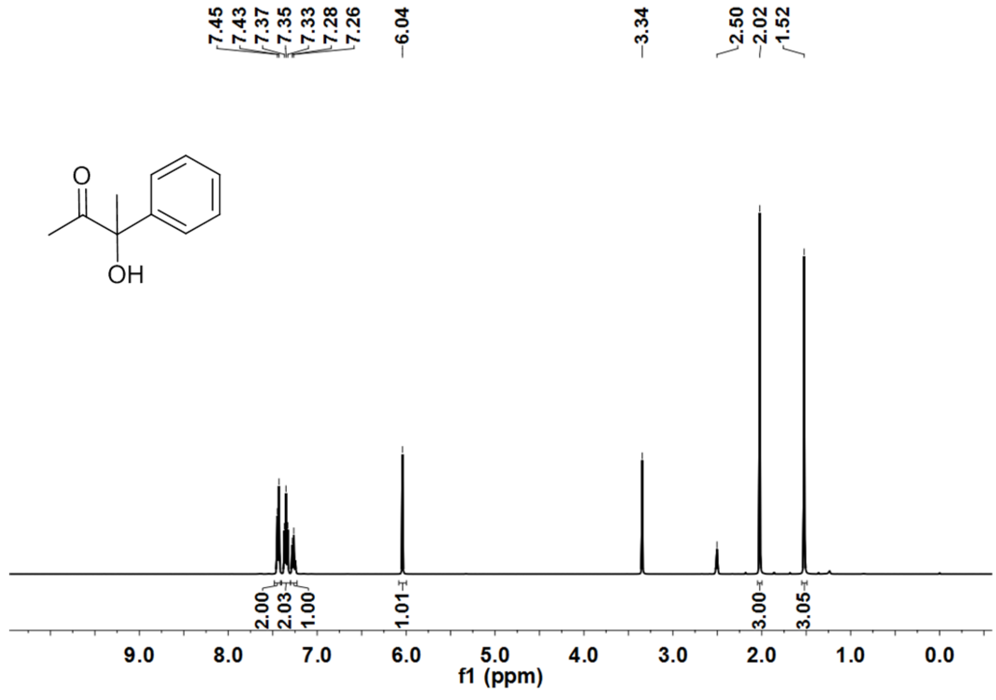

Figure S20. ^{1}H NMR spectrum of 3-hydroxy-3-phenyl-butan-2-one

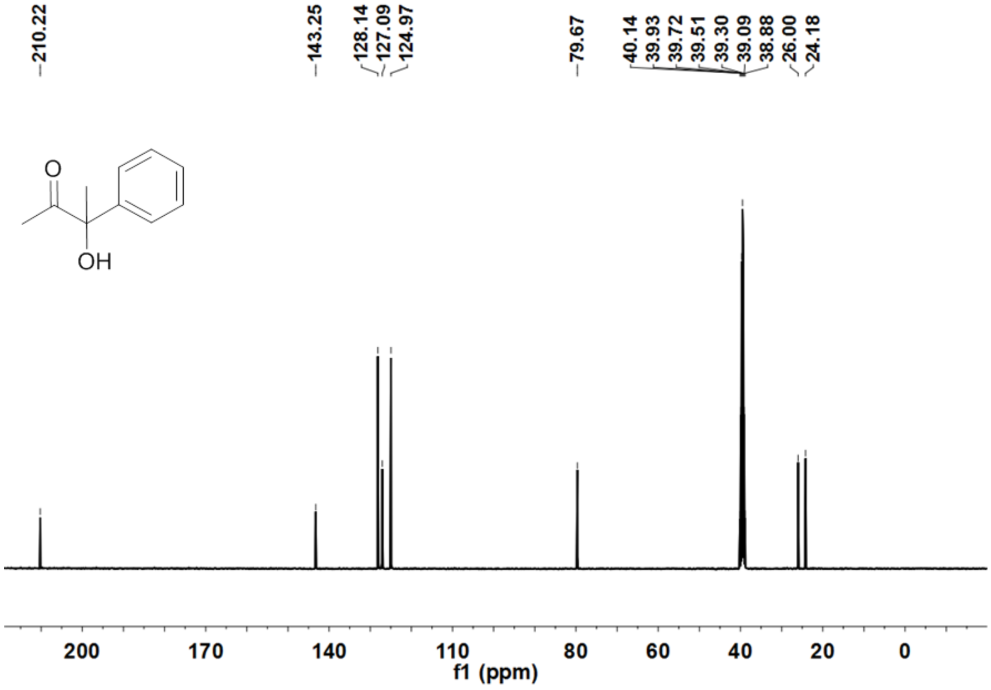

Figure S21. ^{13}C NMR spectrum of 3-hydroxy-3-phenyl-butan-2-one

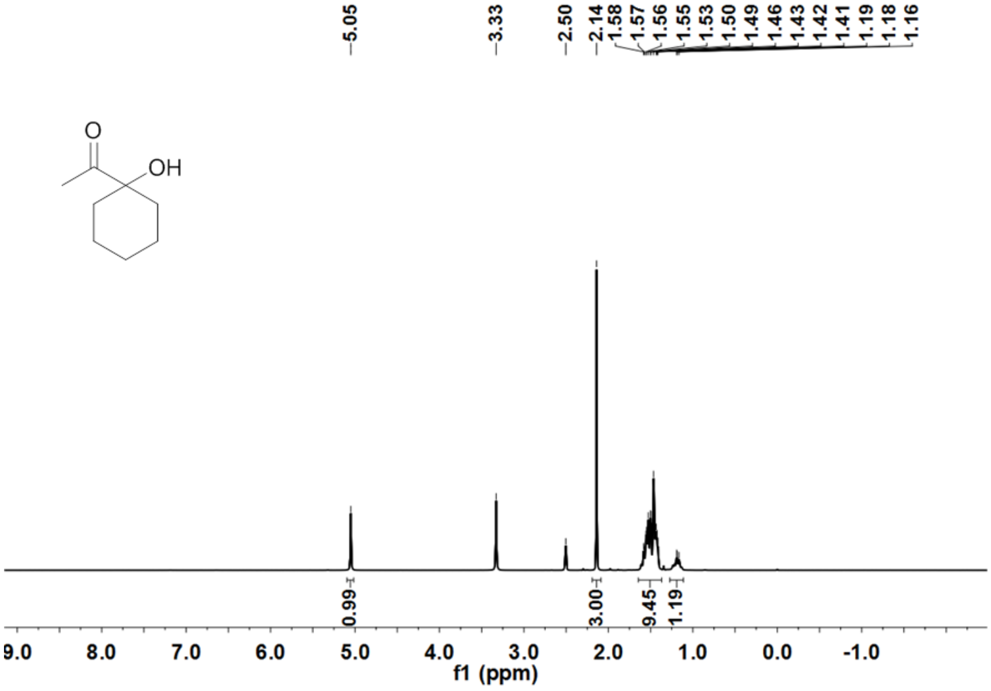

Figure S22. ^{1}H NMR spectrum of 1-acetylcyclohexanol

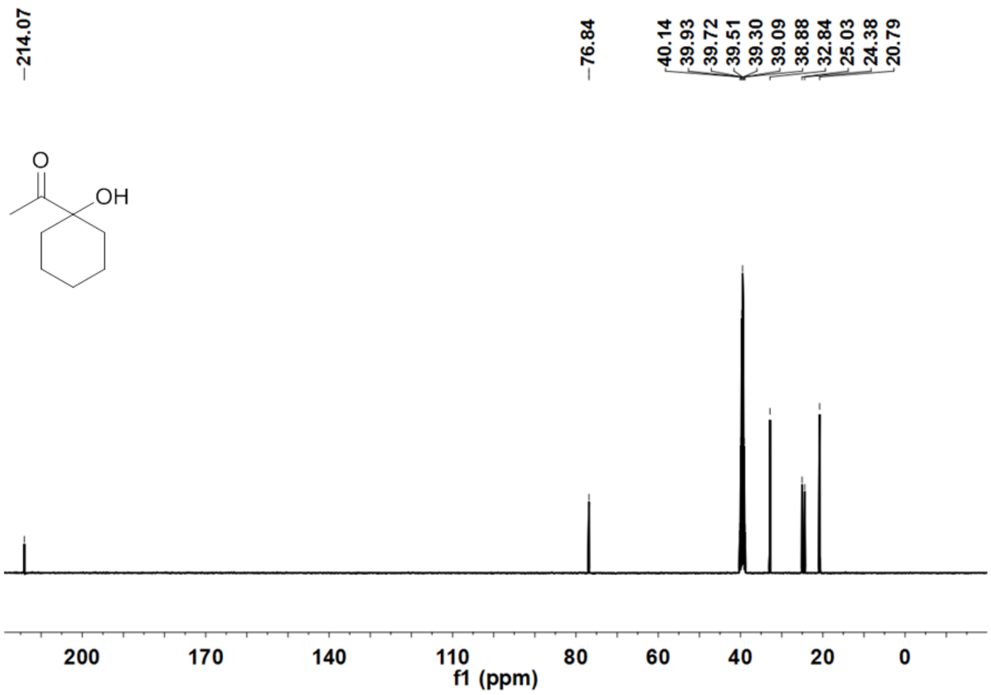

Figure S23. ^{13}C NMR spectrum of 1-acetylcyclohexanol

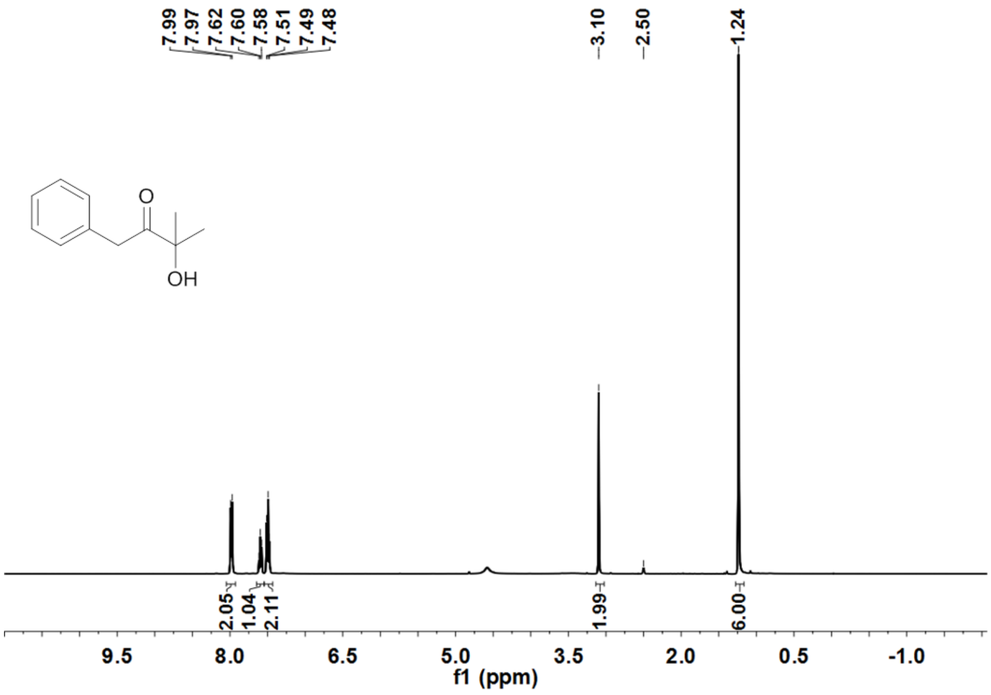

Figure S24. ^{1}H NMR spectrum of 3-hydroxy-3-methyl-1-phenylbutan-2-one

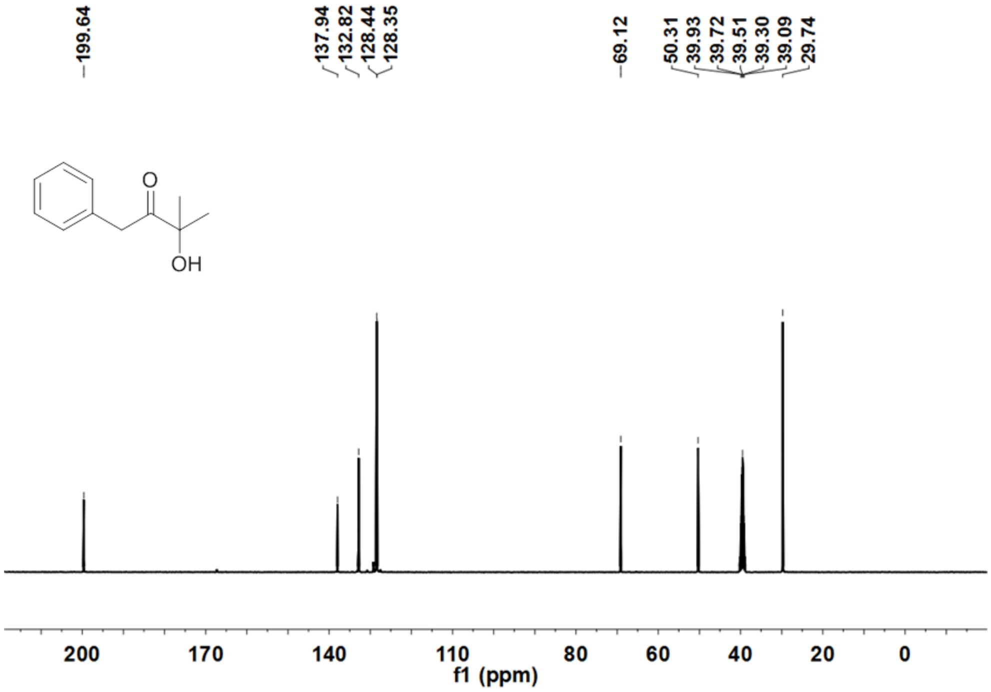

Figure S25. ^{13}C NMR spectrum of 3-hydroxy-3-methyl-1-phenylbutan-2-one

6. References

[S1] a) Y. Tsuji, T. Mizumo, H. Ohno, *Chem. Commun.*, **2011**, 47, 3132; b) C. M. Wang, X. Y. Luo, H. M. Luo, D. E. Jiang, H. R. Li, S. Dai, *Angew. Chem. Int. Ed.* **2011**, 50, 4918.

[S2] X.W. Chen, X.H. Li, H.B. Song, Y. Qian, F. R. Wang, *Tetrahedron Lett.*, **2011**, 52, 3588.
